# Rational In Silico Design of Molecularly Imprinted Polymers: Current Challenges and Future Potential

**DOI:** 10.3390/ijms24076785

**Published:** 2023-04-05

**Authors:** Soumya Rajpal, Prashant Mishra, Boris Mizaikoff

**Affiliations:** 1 Department of Biochemical Engineering and Biotechnology, Indian Institute of Technology Delhi, New Delhi 110016, India; rajpal.soumya@dbeb.iitd.ac.in (S.R.); pmishra@dbeb.iitd.ac.in (P.M.); 2 Institute of Analytical and Bioanalytical Chemistry, Ulm University, Albert-Einstein-Allee 11, 89081 Ulm, Germany; 3 Hahn-Schickard, Sedanstraße 14, 89077 Ulm, Germany

**Keywords:** molecularly imprinted polymers, MIP, computational design, molecular dynamics, quantum mechanics, polymer simulations, machine learning, ML, artificial intelligence, AI, monomer screening, material design, rational design, data analysis

## Abstract

The rational design of molecularly imprinted polymers has evolved along with state-of-the-art experimental imprinting strategies taking advantage of sophisticated computational tools. In silico methods enable the screening and simulation of innovative polymerization components and conditions superseding conventional formulations. The combined use of quantum mechanics, molecular mechanics, and molecular dynamics strategies allows for macromolecular modelling to study the systematic translation from the pre- to the post-polymerization stage. However, predictive design and high-performance computing to advance MIP development are neither fully explored nor practiced comprehensively on a routine basis to date. In this review, we focus on different steps along the molecular imprinting process and discuss appropriate computational methods that may assist in optimizing the associated experimental strategies. We discuss the potential, challenges, and limitations of computational approaches including ML/AI and present perspectives that may guide next-generation rational MIP design for accelerating the discovery of innovative molecularly templated materials.

## 1. Introduction

For the purpose of analysis, purification, or diagnostics, complex mixtures are separated using, e.g., chromatographies or specific antibodies with molecularly imprinted polymers (MIPs) as a synthetic receptor alternative. MIPs are designed affinity materials that can be chemically synthesized such that predetermined specific recognition properties are entailed. Initially, the synthesis strategy was based predominantly on empirical processes involving polymer formation via functional monomers, cross-linkers, and polymerization initiators in the presence of a target template, which could then interact either via covalent or non-covalent bonds. The template is finally extracted to create ‘memory’ cavities within the polymer that resembles the template in functionality and potentially also in shape and size [1]. Since the cleavage of covalent bonds is non-trivial without potentially affecting the binding sites, non-covalently prepared MIPs will be the focus of the present review. However, obtaining robust pre-polymerization complexes is essential during non-covalent imprinting for ensuring high affinity, selectivity, and binding capacity of the resulting MIPs [2]. Nowadays, this rather random process has advanced toward computationally predictable synthesis strategies with a high probability of success, yielding rationally tailorable smart polymer structures. In particular, the past two decades have seen MIP research evolving from ‘trial and error’ towards rational design based on molecular simulations for reducing efforts in experimentally optimizing stoichiometry, monomer selection, and polymer synthesis conditions [3]. The utility of MIP modelling has certainly increased, with almost unlimited access to computational power and sophisticated software packages, and it places tailor-made synthetic recognition materials with high specificity and optimized performance within reach. Hence, the present review aims at summarizing rational design strategies assisting this process for facilitating next-generation materials and methodologies via in silico design and modelling.

Notwithstanding, it should be noted that there has been tremendous progress in MIP technology and materials, with state-of-the-art applications in environmental analysis, food quality, and safety monitoring, and in the health sector, including biomedical and clinical scenarios, owing to their inherently high stability, versatility, and potentially low-cost production [4,5,6,7,8,9,10,11,12,13]. Indeed, performance improvements are noticeable based on an advanced understanding of the physical mechanisms underlying MIP formation and MIP-target recognition via the analysis of each stage during the synthesis. With such insight derived from the advanced analysis of such materials, this in turn stimulates computational modelling, especially of the initial processes occurring in pre-polymerization mixtures and during polymerization reactions, leading toward ‘rational MIP design’ [14].

The most commonly used computational approaches—independently or combined—to tailor the recognition properties of MIPs are quantum mechanics (QM), molecular mechanics (MM), and molecular dynamics (MD) simulations [3,15,16]. These allow for the smart selection of components for polymerization mixtures and consider their interactions affected by physical parameters; finally, one may even derive the binding capacity of the resulting polymer. However, while MIPs are ideally tailored to exhibit selective binding characteristics, due to an intrinsic property of polymers acting as adsorbents, they can lead to non-specific interactions [17]. Several reviews thoroughly discuss the application of QM, MM, and MD to model the fabrication of imprinted polymers [3,15,16,18]. Therefore, herein these techniques will only be briefly outlined.

Primarily, MIP modelling hinges on the basic thermodynamic Equation (1) describing the binding events in templated systems as [19,20]:

∆G_bind_ = ∆G_t+r_ + ∆G_r_ + ∆G_h_ + ∆G_vib_ + ∑∆G_p_ + ∆G_conf_ + ∆G_vdW_ (1)

where the Gibbs free energy change for complex formation (∆G_bind_) is the combined energy change associated with the loss of translational and rotational freedom (∆G_t+r_), restriction of rotors upon complexation (∆Gr), hydrophobic interactions (∆G_h_), residual soft vibrational modes (∆G_vib_), the sum of interacting polar group contributions (∑∆G_p_), adverse conformational changes (∆G_conf_), and unfavorable van der Waals interactions (∆G_vdW_).

This approach maps the situation in a ‘pre-polymerization mixture’ where monomers and target template are mixed to assess these interactions and estimate the energy of the system. In silico, this can be executed as an electronic structure calculation using ab initio, semi-empirical and/or density function theory (DFT) that is included in QM methods. These are highly accurate for a limited number of atoms; with an increasing number of constituents, the disadvantages of QM include the need for extensive computational resources and time. QM relies on the electronic distribution for estimating the total energy of the system. Based on Schrödinger’s equation and several approximations including the decoupling of electronic motion from the nuclear motion (Born–Oppenheimer approximation), the total energy is calculated as the sum of nuclear energy (electrostatic repulsion) and electronic energy, whereby the latter comprises kinetic, potential, and electron–electron repulsion. Principally, ab initio calculations involve the use of different theories, such as Hartree–Fock (HF) theory or Møller–Plesset theory, that approximate the electronic wavefunction, as opposed to density functional theory (DFT) that approximates the Hamiltonian operator [3,21]. These theories vary in the way the correlation effects for electronic motions are incorporated in the calculations. Studies involving ab initio calculations do not appropriately consider basis functions for system-energy calculations, which leads to an overestimation of the true value for intermolecular interactions [22]. This is known as the basis set superposition error (BSSE) that needs to be corrected in QM-based MIP design; yet, only few studies have reported this correction [23,24,25]. Semi-empirical methods are simplified quantum methods and relatively fast in their execution; however, they operate by considering only the valence electrons of the system and parameters derived from experiments.

Electronic motion may characterize intermolecular interactions; however, once the observed system is large, e.g., entailing many template molecules, proteins, etc., molecular mechanics methods that estimate the energy as a function of nuclear positions only are advantageous. The calculations are based on the stretching of bonds or rotation around single bonds using a variety of force-field methods. The most common force fields applied are AMBER, OPLS, and CHARMM [26]. MM therefore allows the simulation of macromolecular systems with less time and computational power; however, this is at the expense of accuracy.

Molecular dynamics comes into play if template–monomer interactions have to be modelled over a period of time to provide insight into the robustness and the dynamics of the interactions. Thereby, the nature of the solvent, temperature, and salt conditions may also be manipulated. MD simulations provide insight into the solvation effect by explicitly adding desired solvent molecules to the system. Alternatively, several quantum methods such as the polarizable continuum model (PCM) may also (implicitly) introduce solvents as a perturbation of the gas-phase behavior of the system to indicate such effects [27].

Although highly accurate, the standalone use of QM methods—for modelling multiple copies of monomers, templates, and explicit solvents—cannot incorporate all parameters and concentrations to emulate real-world experimental conditions, leading to a compromise by limiting the size of combinatorial screening and focus on a selected set of MIP formulations based on experimental knowledge, albeit with the evident limitations of being trapped in a local optimum. Alternatively, QM-MM, QM-MD, MM-MD, and QM-MM-MD combinations are nowadays being increasingly adopted for evaluating multi-molecular systems. These are differentially used according to the template of interest, its properties, the dimensions of monomer libraries to be screened, etc. Herein, we therefore review the use of in silico methods at every step along the MIP synthesis under the consideration that a rigorous and standard modelling approach of imprinted polymers is still not yet established. For highlighting progress in the field, selected relevant studies will be discussed that point beyond the present limitations and toward a more comprehensive theoretical treatment of MIPs. Current limitations will be contrasted by future prospects derived from a multidisciplinary approach to this topic for revolutionizing the rational in silico design of next-generation MIPs.

## 2. Computational Modelling during Polymerization

**Figure 1:**
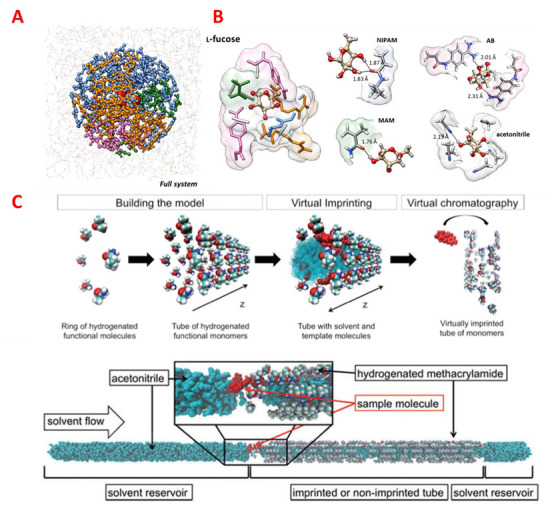
(**A**) Structure of a representative pre-polymerization system used as an input model for MD simulations. The template (spheres representation) is located at the origin of the simulated system and surrounded by a spherical shell of 48 functional monomers (*N*-isopropylacrylamide (NIPAM), pink; acrylamidophenyl(amino)methaniminium acetate (**A**,**B**), orange; methacrylamide (MAM), green, 48 cross-linker units (ethylene glycol dimethacrylate (EGDMA)) blue; and an explicit solvent box (acetonitrile), gray. (**B**) Examples of prepolymerization complexes and hydrogen-bonding interactions observed with target template (l-fucose). Reprinted with permission from [36], Copyright 2021, American Chemical Society. (**C**) Virtually imprinted tubes templated with 17-β-estradiol demonstrate the concept for virtual chromatography experiment simulations;. The template is represented by the red molecule, the functional monomers by the colorful molecules and the solvent by the cyan molecules. Reproduced from [37] with permission from the Royal Society of Chemistry.

MIP optimization practically involves six major steps (1–6), and the intervention with computational methods takes place either individually at each step or for multiple steps together (Figure 1). Ideally, for the closest prediction of the experimental performance, all these steps require simulations in a given order; however, only few studies report on such a comprehensive approach. Nevertheless, studies that have developed MIPs based on predictive modelling of at least two or three steps are highlighted in Table 1. The experimentally synthesized MIPs based on computational methods show significant binding characteristics in terms of their imprinting factor (IF) and binding capacity (Q). This table provides insight into how computational research is expanding for MIP development; yet, only few studies explore all the steps listed below:

1. Appropriate monomer(s) selection;
2. Monomer-to-template ratio optimization;
3. Monomers, template, and additional polymerization conditions/agents analyzed at different solvent conditions;
4. Structural polymer establishment and optimization;
5. Polymer-template interactions (generic);
6. Polymer-template interactions in a target solvent and binding to structural analogues for evaluating selectivity.

Simulating a structural polymer itself is computationally expensive; if the template is a large species (e.g., protein, etc.), the last step (6) significantly adds to the computational load. Generally, two or three steps are predictively optimized in most studies, while the remaining characteristics are experimentally tested and optimized (Table 1). However, increasing access to advanced computational tools and softwares has enabled combined applications of electronic structure calculations, MD simulations, and multivariate data analysis strategies to all aspects of MIP design and synthesis.

Typically, the first three steps are collectively referred to as the pre-polymerization mixture optimization (Figure 1A,B). It is well known that the nature and extent of non-covalent interactions including electrostatic, hydrophobic, and H-bond interactions between the monomer and template dictate the recognition properties of the final MIPs. This has been investigated both with experimental [28,29,30] and theoretical approaches [31,32,33,34,35]. The next steps are related to the actual polymerization process, the establishment of the structural polymer, and post-polymerization assessment (Figure 1C). These steps concerning the resulting polymer morphology and the influence of the morphology on recognition have been explored less frequently to date (step 4, 5) [35].

**Table 1:** Molecularly imprinted polymers designed with computational intervention at multiple steps using quantum mechanics (QM), molecular mechanics (MM), molecular dynamics (MD) methods, and combinations of QM-MD, MM-MD, QM-MM, and QM-MM-MD.

Method Used	Steps Explored	Monomers Used/Screened	Computational Technique	Template	MIP Performance	References
QM	1,2	Pyrrole, 3,4-ethylenedioxythiophene and m-phenylenediamine	Semi-empirical PM3, DFT method at B3LYP/6-31+G level	Sulfamethizole	IF: 8.4 LOD ~ 1.7 nM	[38]
	2,3	MAA	DFT method at B3LYP/6-311G (d) level; PCM for solvent effect	Naltrexone	Q: 11.60 mg/g, IF: 2.27	[39]
	2,3	Pyrrole	DFT method at B3LYP/6-311+G*; PCM for solvent	Dopamine	LOD ~ 10 nM	[22]
	1,2,3	AA, MAA, AAM, MMA, TFMAA, p-VBA, *N*-vinyl pyridine, allyl alcohol, 1-vinylimidazole	Semi-empirical PM3, DFT method at B3LYP/6-31G(d,p); PCM for solvent effect	Metaproterenol	LOD ~ 0.01 µg/mL; IF: 5.2	[40]
	1,2,3	AA, MAA, MAAM, 2-VP, STY, allylamine	DFT method at B3LYP/6-31G+ (d, p) level; PCM for solvent effect	(S)-warfarin	IF: 25.7; Recovery ~ 90%	[41]
	1,2,3	AA, MAA, AAM, MAAM, MMA,4-VP	HF 6-311G^**^ basis set, DFT method at B3LYP/3-21G level PCM for solvent effect	Metformin	LOD ~ 0.005 ng/mL; Recovery ~ 99%	[42]
	1,2,3	AA, MAA, AAM, 4-VP, 1-vinylimidazole, 4-vinylimidazole	DFT method at B3LYP/6-311G (d,p) level; PCM for solvent effect	Cannabinoids	–	[43]
	1,2,3	AA, MAA, TFMAA	DFT method at B3LYP/6-31G (d,p) level; PCM for solvent effect	Tramadol	–	[44]
	1,2,3	AA, MAA, AAM, MAAM, TFMAA, ITA, p-VBA, 2-VP, 4-VP, acrolein	DFT method at B3LYP/6-31++G (d,p) level (also, second-order Møller–Plesset (MP2)); PCM for solvent effect	L-Serine	–	[23]
	1,2,3	MAA, AAM, ITA, VP	DFT method at different levels: B3LYP, BHandHLYP, M062X, and ωB97xD and basis sets: 6-31G(d,p), 6-31++G (d,p) (Comparative study); PCM for solvent effect	2,3,7,8-tetra-chlorodibenzo-p-dioxin	Q: 3.7 mg/g, IF: 2.371	[25]
	1,2,3	AA, MAA, TFMAA, p-VBA	DFT method at B3LYP/6-311G (d,p) level; PCM for solvent effect	Dinotefuran	Recovery: 89.87 ± 4.64%	[45]
	1,2,3	MAA, AAM, vinyl benzene	DFT method at B3LYP/6-311++G (d,p) level; PCM for solvent effect	3-hydroxy-2-methyl quinoline-4(1H)-one (HMQ), dummy template	Q: 5.21 mg/g, IF: 6.43	[46]
	2,3,4,5 6	MAA	Semi-empirical PM3, DFT method at B3LYP/6-31G level	Hydroxyzine, cetirizine	–	[47]
MD	2,3	MAA, MMA	Amber99, GAFF	Bupivacaine	–	[35,48,49]
	2,3	4-VP	COMPASS	4-nitrophenol	IF: ~1.4	[50]
	1,2,3	MAA	Amber99, GAFF	17-β-estradiol	–	[51]
	1,2,3	MAA, poly (ethylene glycol) ethyl ether methacrylate	Amber ff14SB, GAFF	MMP9 protein	IF: 1.3	[52]
	4,5,6	MAA	OPLS-AA	Cholesterol	–	[53]
	1,2,3,4, 5,6	MAA, AAM, *N,N*′-methyl- enebisacrylamide and 2- (dimethylamino)ethyl methacrylate	MARTINI ff, OPLS-AA, coarse-grained (CG) lattice, Monte Carlo (MC) simulations	Lysozyme and cytochrome c	IF: ~1.2	[54]
QM-MD	1,2,3	MMA, MAAM, 2-VP, 4-VP	DFT method at B3LYP/6-31+G (d,p) level; PCM for solvent effect; COMPASS	5-(3,5-Dichloro- 2-hydroxybenzyl amino)-2-hydroxybenzoic acid	–	[55]
	1,2,3	1-(triethoxysilylpropyl)-3 -(trimethoxysilylpropyl)-4,5-dihydroimidazolium iodide; 4-(2-(trimethoxysilyl)ethyl)pyridine; 1-(3-(trimethoxysilyl)propyl)urea	DFT method at B3LYP/6-311+G(2d,2p)//HF/6-31 G* level; (also tested (B3LYP, CAM-B3LYP, LC-wPBE) with different basis sets (6-31++G(d,p), 6-311++G(2d,2p), cc-pVTZ); PCM for solvent effect; OPLS-aa	Naproxen	–	[56]
	1,2,3	*N-*allyl thiourea, *N*-Benzoyl thiourea, (2, 6-difluorophenyl) thiourea, 1- (3-carboxyphenyl)—2-thiourea, 1-Benzoyl-3-(2-Pyridyl)− 2-Thiourea	DFT method at B3LYP/6-31+G (d,p) level; PCM for solvent effect; universal force field (Material studio)	H3AsO3 (Heavy metal)	–	[57]
	2,3,4	Cyt-S4: cytosine-bis(2,2′-bithienyl)-(4-carboxyphenyl)methane ester	DFT method at the B3LYP/3-21G^*^ level; PCM for solvent effect; OPLS	6-Thioguanine	IF:2.9; LOD: 10 µM	[58]
	4,5,6	MAAM	HF/PM3; GAFF	17-β-estradiol	–	[37]
MM-MD	1,2,3	28 monomers including 2-hydroxyethyl methacrylate, MAA, ITA	SYBL PACKAGE	Curcumin, ephedrine	IF: 1.3–2.0	[59,60]
	1,2	AA, MAA, AAM, MAAM, 2-VP, 4-VP	CHARMm and MMFF94	Amlodipine	Q: 53.77 µg/mg; IF: 2	[61]
	2,3,4,5	MAA	PCFF, Monte Carlo (CMC) simulations	Caffeine, theophylline	–	[62]
	1,2,3	AA, MAA, TFMAA, ITA, 2-VP, 4-VP, STY, 2-acrylamido-2-methyl-1-propanesulfonic acid, allylamine, 1-vinylimidazole, *N*,*N*-diethylamino ethyl methacrylate acrylamide, 2-hydroxyethyl methacrylate	SYBL; AMBER99sb force	Melamine	IF: 1.39	[63]
	1,3	MAA, ITA, NIPAM, *N*-hydroxyethyl acrylamide, *N*-phenylacrylamide, 2-acrylamido-2-methyl-1-propanesulfonic acid	GAFF, GROMOS96	SARS-CoV-2 spike protein epitopes	–	[64]
QM-MM	1,2,3,4,5	AA, MAA, TFMAA, 4-VP, allylamine, 1-vinylimidazole, 2-hydroxyethyl methacrylate	DFT method at the B3LYP/6-311+G(d,p) level; PCM for solvent effect; CHARMm	1-(2,4-Difluorophenyl)-2-(1H-1,2,4-triazol-1-yl)ethanone	Q: 0.333 µmol/g	[65]
	2,3,4,5,6	MAA	DFT method at the B3LYP/6-311+G(d,p) level; PCM for solvent effect; CHARMm	Tyramine	IF: 4.27; Recovery: ~95%	[66]
	2,3,4,5,6	MAA	Semi-empirical PM3; conductor-like screening model (COSMO) for solvent effect; GAFF	Histamine, l-histidine and d-histidine; theophylline, caffeine, and theobromine	–	[67]
	1,2,3	MAA, AAM, MMA	HF/6–31G(d); PCM for solvent effect; MMFF94x	6-mercaptopurine	Q: 0.822 mg/g, IF: 3.99	[68]
QM-MM-MD	1,2,3,4,5,6	AA, MAA, AAM, TFMAA, ITA, 4-VP, isopropenylbenzene, 2-hydroxyethyl methacrylate, 2-(diethylamino)ethyl methacrylate, allylamine	DFT method at the B3LYP/6-311+G(d,p) level; CHARMm	Octopamine	IF: 6.37	[69]

Abbreviations: Monomers: AA: acrylic acid; MAA: methacrylic acid; AAM: acrylamide; MAAM: methacrylamide; MMA: methyl methacrylate; TFMAA: trifluoromethacrylic acid; *p*-VBA: *p*-vinyl benzoic acid; STY: styrene; 2-VP: 2-vinyl pyridine; 4-VP: 4-vinylpyridine; ITA: itaconic acid; NIPAM: *N*-isopropylacrylamide; QM methods: DFT: density functional theory; HF: Hartree–Fock; PM3/6 (semi-empirical): parameterized model 3/6; B3LYP: Becke three-parameter exchange-correlation functional; PCM: polarizable continuum mode; MM force fields: OPLS-AA: optimized potentials for liquid simulations all-atom); GAFF: general AMBER force field; AMBER: assisted model building with energy refinement; CHARMM: Chemistry at HARvard Macromolecular Mechanics, GROMOS: GROningen MOlecular Simulation; COMPASS: condensed-phase optimized molecular potentials for atomistic simulation studies; PCFF: polymer consistent force field; MMFF94x: Merck molecular force field; Q: binding capacity; IF: imprinting factor; LOD: limit of detection.

## 3. In Silico Monomer Selection

The first step of computational optimization to guide experimental design is the selection of suitable monomers that can interact with a target template, preferably through non-covalent interactions such as electrostatic, van der Waals, and H-bond interactions. The transition from testing a small set of monomers to in silico screening of large monomer libraries has been beneficial for the MIP development process by speeding up the exploration of novel monomer functionalities [70,71,72,73,74,75,76,77]. Among these libraries, commonly used monomers include acrylate derivates such as AA, MAA, AAM, MAAM, MMA, and TFMAA that can offer diversity of functional groups to uniquely interact with a target template. Numerous MIP related articles continue to employ these limited monomers, thereby restricting the potential of the computational resources and leaving out the remaining class of monomers (Table 1). Generally excluded are alkoxysilane-based monomers that can form smart, sol-gel materials with high thermal stability, water compatibility, and easier control of porosity and thickness in the resulting ‘molecularly imprinted xerogel’ [78]. These monomer species are greater in size, implying an increased number of atoms to input to computational modelling. A few studies employed a simplified version of a QM model for alkoxysilanes to keep reasonable computing time while choosing a relatively modest initial basis set 3-21G [21]. Others have combined QM, MM, and MD studies for faster assessment of the non-covalent interactions [56,79,80,81,82]. In order to reliably predict best performing monomers, MM/MD-based ‘fast-screening’ determining a few monomers for a QM-based ‘thorough screening’ is a fair interplay of both techniques [71].

While extensive research is available for acrylate-based polymers to establish that the strength of monomer-template interactions translates to high efficiency MIPs, this needs to be thoroughly explored for molecularly imprinted xerogels, the main aspect being the active hydrolytic-polycondensation reactions relevant to silane monomers varying in different solvents and temperature [83]. This can affect the pre-polymerization complex formation between the template and monomers where the silane can variably exist in its native, hydrolyzed, and condensed state. With the advent of computational approaches such as quantum Monte Carlo or metadynamics, an improved and elaborated simulation may be possible [82].

Furthermore, there is a requirement for a definitive protocol to obtain suitably ranked monomers for different target templates (including proteins) without the need for further experimental validation. Two studies have devised a generic MM/MD method; however, it is applicable mostly to low molar mass templates [84,85]. For protein imprint optimization, the employment of MM-based force fields has been varied by different research groups [26,86,87,88,89]. It was noted in a comparative study that one force field might be more reliable than the other for a template to assess monomer–template interactions [90]. A lack of a universal protocol leads to an arbitrary selection of computational methods, and so the predictions need to be continuously validated with some degree of experimental optimization. This can inspire artificial intelligence/machine learning to engage different methods and comprehend available research findings to develop an all-inclusive protocol employable for all monomers and templates.

Nevertheless, the current methods can greatly reduce the tedious process of experimental screening. Virtual libraries of more than 60–70 commonly used monomers can be possibly generated and screened [91]. Simultaneously, practical problems associated with MIP application need to be contemplated. For example, high-cost monomers cannot be used for large scale synthesis serving as solid-phase sorbents, and so virtual libraries can be grouped into high and low-cost monomers. Another possible grouping can be made for biocompatible monomers that are relevant for *in vivo* applications [92]. Monomers libraries can include information about the porogens with which they are compatible, as well as whether they can function together with a target template. Later sections also highlight the need for water compatible monomers, especially for biomolecule imprinting. As the existing monomer library comprises either insoluble or partially water-soluble monomers, the list can be expanded with bio-based monomers such as gelatin, chitosan, cellulose, sodium alginate, and cyclic oligosaccharides. These can form a hydrophilic exterior to be compatible with an aqueous medium and a hydrophobic core rearrangement to interact with organic moieties during molecular imprinting [93].

Next in the category are metal ions, such as Ni^2+^, Zn^2+^, Cu^2+^, and Co^2+^, that can form coordination complexes with proteins, peptides, and His-tagged oligonucleotides with high affinity [94,95,96]. For example, Ni^2+^ was used with a C-terminal nonapeptide of human serum albumin for imprinting the peptide [97]. Metal ions possess the ability to generate Lewis acid sites in the polymer network, act as a functional monomer, and reversibly coordinate with small organic molecules [98]. The advancing field of polymer research can innovate the library of monomers, and their efficiency can be evaluated for MIP design.

## 4. In Silico Template Modelling and Selection

The size of the template is the main determinant for the predictive method to be used. Lower mass templates (MW<1000 g/mol) can be modelled using QM methods; however, these are difficult to apply for large templates, such as proteins. An increase in the size and complexity of the template can introduce binding site heterogeneity in the respective MIP cavities [99,100]. To model large templates (proteins, peptides, nucleic acids, etc.), MM/MD methods can be used that mimic the searching algorithms applied in drug design [16]. MM methods involve docking to indicate preferential monomer binding sites on the protein surface. However, it is not a straightforward process, as it is for small templates, for the following reasons. Firstly, the simulations require the template protein in the form of its crystal structure data, which are mostly acquired from the protein data bank (PDB), but those proteins where crystal structure data are not available have to be modelled *a priori*. Secondly, multiple binding sites have to be assessed for their interaction energy with the monomer. As the solvent accessible surface area (SASA) of the protein is composed of both hydrophilic and hydrophobic residues, analogous monomers are used in screening. Monomers with an ability to engage several binding sites are ideal for molecular imprinting [86,87].

Experimentally, the combinatorial use of multiple monomers inculcating diverse functionalities has been shown to improve specificity in polymer cavities [101,102]. To mimic this, multi-monomer simultaneous screening strategies can be employed to select from a possible combination of monomers and can also parallelly account for monomer–monomer interactions [90]. Thirdly, proteins are predisposed to conformational change due to monomer-induced protein instability, and so molecular dynamics studies are required to predict the conditions including (multiple) monomers, solvent, time, and temperature that can maximally preserve the protein structure during imprinting. This step is crucial in guiding the fabrication of high-performance MIPs but remains computationally challenging with respect to the power and resources required. Modelling an entire pre-polymerization complex is expected to simulate high monomer to protein molar ratios of up to 2000:1 as is commonly seen in the literature [26]. Therefore, predictive design for templating proteins is mostly limited at the screening stage as opposed to the possibility of the simulation of most steps for small molecule imprinting (Table 1) [25].

During MIP synthesis, the large-sized proteins have a higher tendency to entrap inside the bulk polymer or to undergo incomplete template removal, denaturation, etc., and therefore, advanced strategies for imprinting have been adopted such as surface imprinting and solid-phase imprinting; harsh and lengthy polymerization conditions have been minimized; and, more importantly, the use of complete proteins as a template has been substituted with smaller fragments and/or epitopes [100,103,104,105].

Theoretical calculations for epitopes and fragments can be easier to conduct compared to protein–monomer interaction modelling. Additionally, required epitope design and selection can be guided from previous studies [106]. Fundamentally, peptides and/or fragments have the potential to form a structural identity in a solution that can be different from that native to the protein [94], so modelling of structural characteristics in solution may be essential based on the application. Similarly, the structure of the epitope can also be affected by monomer-induced interactions. In any event, the multi-factor modelling for smaller fragments/epitopes is possible in a computing time less than that for whole proteins.

A related analogue or ‘dummy’ templating approach is also available for lower mass template, especially when it is expensive or difficult to acquire in sufficient amounts [46]. The computational intervention (as seen by Wyszomirski and Prus) can allow faster comparison of the monomer affinity of dummy templates with actual templates [107]. Monomers that have higher affinity for both the templates instead of preferring one to the other can be selected, and the tedious trial-and-error methods can be superseded. Subsequently, this may be simulated at a polymer level to evaluate the shape and size complementarity of the templated cavities with the actual template. MD-based polymer simulations can potentially map structural differences in MIPs in case they are non-specific to the original template.

Template size can be as large as biological organisms such as bacteria, viruses, and yeast cells that have been employed for imprinting to develop diagnostics for direct pathogen binding [108,109,110,111,112,113]. Bacterial/viral imprinting is currently in its initial stage for multiple reasons such as difficulty in the handling of pathogenic bacteria as a template, the large size of the organism leading to non-specific binding sites, and high resource requirements for simulating such complex systems. Advancing predictive models are available for cell membrane components of bacteria [114], and their structural data may be employed to simulate MIP development. However, the branch of whole cell imprinting still remains highly challenging.

## 5. In Silico Solvent Modelling

**Figure 2:**
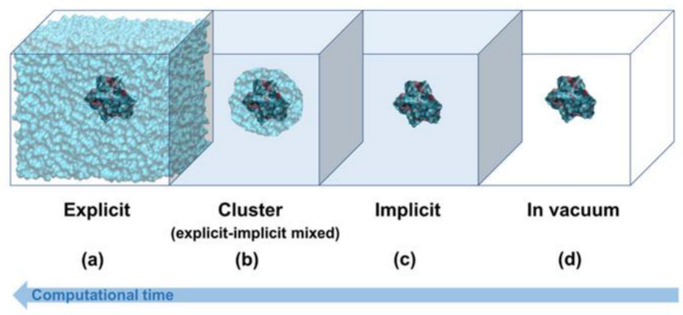
Schematic representation of solvent models used to study the effect on the complex. Three different solvation models—implicit (or continuum), cluster/continuum (or hybrid implicit/explicit), and fully explicit (**a**–**c**)—can be used to simulate complex interactions and compared with those in vacuum environments (**d**). These simulations are arranged according to the computational time involved. Reproduced from [116] with permission from Springer Nature.

Porogen selection precisely determines the final surface morphology and pore size of the MIPs [115]. As a rule of thumb, the solvent should cause the least interference with the template–monomer (T–M) complex. The choice of solvents can vary from polar solvents (water and acetonitrile) to non-polar solvents (chloroform and toluene) based on compatibility with the polymerization components. Water, being highly polar, is not an ideal solvent, as it generates maximum hydrogen-bond competition with the complex [44,99]. Computational methods allow the visualization of such effects, thereby preventing damage to polymer functions. The solvation effect can be predictively studied by involving QM in an implicit solvent model and MD in an explicit model (Figure 2). However, the choice of implicit and explicit solvent modelling must be rationally incorporated.

Implicit models treat the solvent as a structureless continuum accounting for certain dielectric and interfacial properties, while explicit models describe the physical spatial resolution by the actual addition of solvent molecules to the system. During imprinting, solvent molecules comprise over 90% of the system space, and so explicit modelling is important to assess the number of interacting particles and the number of degrees of freedom; however, most of the MIP simulations only consider implicit solvent modelling (Table 1). Omitting the explicit atomistic description of solvent prevents consideration of hydrogen bonds with solvent, over-stabilized salt bridges and hydrogen bonds within the solute, incorrect ion distribution, and unphysical sampling [117,118,119,120]. On the other hand, due to this absence of viscosity, the conformational search of the T–M can become faster. Ideally, during polymer formation, there are large atomic displacements that can influence the solvation shell, and both the models may therefore be proportioned for a better understanding of the process. Currently, the deciding factor in choosing the method of solvent simulation relies on the system size, and with an increase in size, there is a noticeable shift from implicit to explicit modelling.

Ideally, the medium should be compatible (miscible) with the polymerization mixture components. Most studies demonstrate use of non-polar, aprotic porogens that can interact less but dissolve the T–M to create a strong imprinting effect [121]. There are few templates that require a polar environment, for example, biomolecules (particularly proteins) that exhibit a conformational stability in aqueous solvents. The high polarity of the solvent greatly affects the T–M complex, which necessitates solvent modelling. With the large size of system, this is possible with MD simulations through the explicit addition of water molecules. Since implicit modelling has mostly translated to accurate predictions for small molecular templates, it will be interesting to combine it with protein imprint simulations; however, the use of both models requires higher computational capacities.

An alternative derived from the epitope/peptide imprinting approach can be extensively explored with the current computing potential. Notably, peptides can be designed to be soluble in organic solvents that can enable flexible solvent selection that minimally disrupts the T–M interactions. Comparing peptides with proteins, the size of computational simulations can be reduced, and the accuracy of predictions can be readily improved by combining both implicit and explicit solvent modelling. Some hybrid methodologies may be employed to combinatorially report the density fluctuations of the solvent around the T–M complex (explicit solvation) and give a reasonable description of the solvent behavior (implicit solvation) [71,122] (Figure 2b).

The list of commonly simulated porogens does not include a new class of ionic liquids or greener alternatives such as deep eutectic solvents (DES) and supercritical carbon dioxide (scCO_2_) [94,123,124]. The predictions may be complex for DES as they have several uses in molecular imprinting, not only as porogenic solvents but also as functional monomers, cross-linkers, and modifiers; therefore, their structure needs to be appropriately optimized and modelled [123,125].

## 6. In Silico Polymer Modelling

**Figure 3:**
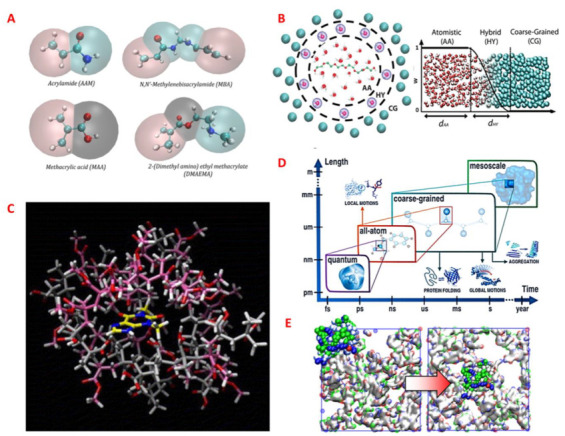
(**A**) Atomistic and CG representation of the hydrogel monomers. Solid beads represent the atomistic model and correspond to carbon, nitrogen, oxygen, and hydrogen in cyan, blue, red, and white, respectively. Translucent beads represent the different types of CG beads (polar, neutral, or apolar). Reprinted with permission from [54], Copyright 2018, American Chemical Society. (**B**) A solvated macromolecule surrounded by water molecules is shown schematically on the left, with the water molecules represented atomistically close to the macromolecule (denoted as AA zone) and in a CG mode that is farther from the macromolecule (denoted as CG zone), with a hybrid (HY) zone in between the AA and CG zones. This transition is shown in the right figure as a function of radial distance to the macromolecule. Reprinted with permission from [130], Copyright 2016, American Chemical Society. (**C**) Atomistic models of copolymer structures constructed using methacrylic acid (MAA) and methylmethacrylate (MMA) and observed for interaction with theophylline (THO). THO molecule is visible inside the binding pocket. Color scheme: THO carbons (yellow), MAA carbons (gray), MMA carbons (pink), oxygens (red), nitrogens (blue), hydrogens (white). Reprinted from [128], Copyright 2006, with permission from Elsevier. (**D**) Application domains for molecular modelling at various resolutions, including quantum, all-atom, coarse-grained, and mesoscale. Approximate time scales and system sizes (lengths) are shown on the plot. By combining tools of various resolutions into multiscale schemes, the application ranges that are covered, can be expanded. Reprinted with permission from [131], Copyright 2016, American Chemical Society. (**E**) Coarse-grained simulation of protein-imprinted hydrogels. Reprinted with permission from [54], Copyright 2018, American Chemical Society.

Majority of the computational MIP research based its predictions on the pre-polymerization mixture to establish experimental correlations [126,127,128]. Although most studies do establish a positive correlation, some have argued that no correlation is shown with binding specificity (but binding capacity) [60]. Extensive simulation of the polymerization step is necessary for the rational design of MIPs. The classical molecular simulations of polymers can be categorized broadly into coarse-grained (CG) and atomistic models. The former can be used to determine the structure/morphology for polymeric systems at a broad range of conditions or polymer design parameters and is based on reducing some degrees of freedom in the system by grouping selected atoms/groups of monomers into a CG “bead” (Figure 3A,B). The latter may be used for the analysis of local monomer structure or the high-frequency motions of individual atoms and elucidation of monomer-level (re)arrangements, fluctuations, or interactions, within a disordered or ordered polymer system (Figure 3C). Atomistic models are comparatively accurate and computationally demanding. These force-field-based methods are system specific and are generally limited to length scales of 1−100 Å and time scales of 1 fs−100 ns [129] (Figure 3D).

Macromolecular simulations based on CG can highlight the events of template aggregation and matrix deformation [132,133,134,135,136], and those based on atomistic detail can present the spatial arrangement of FMs influenced by templates and crosslinkers [37,137,138]. Additionally, morphological differences may be observed in the imprinted sites after solvent removal, for example, when volatile organic solvents are employed [139] or in the case of imprinted hydrogels [54]. It may also be necessary to understand the dynamics in highly rigid MIPs as opposed to lightly cross-linked polymers [135,140].

Predictive analysis for biomolecular imprinting is infrequently used to analyze the behavior of the macromolecular host and template, especially when a research gap already exists in understanding protein behaviors. Srebnik and coworkers moderately explored (globular) protein imprinted polymers using CG-lattice Monte Carlo (MC) simulations [140,141,142]. To resolve finer aspects elucidating the template behavior in the matrix, Zadok and Srebnik additionally combined molecular dynamics (MD) to highlight their static and dynamic behavior in MIPs and NIPs through simulation of the complexation, reaction, swelling, washing, and rebinding (Figure 3E). Simulations are requisite for proteins, as they require a flexible and biocompatible matrix for efficient transport but are expected to generate rigid imprinted sites enabling good selectivity and affinity [54]. Although current contributions have fairly elaborated on the imprint process, there is a lack of extensive analysis to develop highly specific MIPs, which need to match the levels of natural antibodies, especially for proteins.

**Figure 4:**
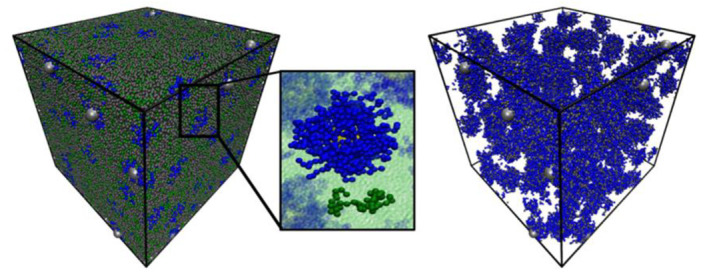
Simulation snapshots of polymer grafted nanoparticles (silver beads for particles and blue beads for grafted polymers) in an explicit homopolymer matrix (green beads). The left image shows all components of the simulation box; the center image shows representative chain configurations for the grafted and matrix chains; and the right image is the simulation box showing only the polymer grafted nanoparticles with all matrix chains hidden. Reprinted with permission from [144], Copyright, 2015, American Chemical Society.

MIP development has mostly been simulated as a bulk polymer, but surface imprinting and solid-phase imprinting are advanced strategies that may follow different dynamics of design. Typically, the proteins/templates are immobilized in both the cases, hence, their surface will be differentially available to the polymerization components. Surface imprinting on nanoparticles as opposed to planar/sensor surfaces can also produce different interaction energies in the complex which have not been studied in silico. For this, a combination of current MIP simulation methods and those generally applicable in polymer sciences can inspire the predictive modelling of different types of imprinting strategies. For example, computational modelling to characterize the behaviors and conformations of polymers on spherical nanoparticles (Figure 4) or nanotubes, as reviewed by Gartner and Jayaraman, can be used [129]. Similarly, MD simulations of polymers developed on substrates to analyze interfacial structure, dynamics, energetics, and mechanical properties can be exploited [143]. This can be extrapolated to study the behavior of MIPs developed with surface imprinting on electrochemically and plasmonically active surfaces and can be used to improve the imprint and sensor performance.

## 7. In Silico Polymer Performance Evaluation

It is abundantly clear that there are numerous parameters that are required to be optimized pre-, post-, and during polymerization, which has encouraged the use of chemometrics involving the application of mathematical and statistical methods to better analyze chemical data. This employs a set of factors, such as type and amount of monomers, cross-linkers, initiator, solvents used for the target template(s), stoichiometric ratios, temperature, and rebinding environment. These factors are used differentially in experimental design methods such as fractional factorial, full factorial, central composite, Box–Behnken, and Doehlert. The gathered data are subjected to statistical analysis such as analysis of variance (ANOVA) and principal component analysis (PCA). Different methods used in the multivariate calibration of data are multiple linear regression (MLR), principal component regression (PCR), partial least-squares regression (PLSR), and artificial neural network (ANN) [3,14].

Chemometric approaches are currently limited to experimental data analysis but can be extended to computational data for better evaluation and predictions. An example headed in this direction is given by Baggiani et al. who employed a semi-empirical AM1 method to obtain 16 molecular descriptors for 52 related phenols and used PCA to compare it with the chromatographic selectivity of a pentachlorophenol MIP for the templated and the non-specific phenols [145]. This indicates the amount of data sets that can be generated and potentially evaluated in a ‘rational’ chemometric approach. Moreover, a comparison of statistical analysis based on experiments and computations can possibly highlight the efficiency of computational optimization at each step.

When MIP selectivity is considered as a factor, testing with non-specific templates is a common practice but may be insufficient to thoroughly establish the MIP performance. Several studies explore all six steps of MIP modelling and simulate the extent of interactions with specific and non-specific templates (Table 1). However, pertaining to MIPs’ application in either environmental, food, or biological systems, the specificity of the template binding may be simulated as part of a complex matrix. Currently, polymer simulations are performed in simple solvents but can be innovatively extended to modelled fluids based on their intrinsic components. For instance, it is necessary to test MIPs for their ability to ultimately bind in human saliva (as such or diluted) when they are developed as artificial antibodies to target an oral biomarker/antigen [146,147,148]. Before actually proceeding to in situ testing, an experimentally simulated artificial saliva can be used [149,150] composed mainly of buffer ions, tween 20, xanthan gum, mucin, and amylase proteins [151], and this in turn can be computationally mimicked by adding the respective atomic and crystal structures to the MD simulation box along with the modelled MIP. Variables such as the matrix viscosity, density, concentration, and overall complexity will also need to be accounted for to precisely predict the performance of the MIP [152].

MIP design can significantly progress with the available computational tools. For better parameterization of the process, more in silico data are required, since most studies have employed chemometrics only through experiments. The detailed understanding offered by multivariate analysis can methodologically improve MIP performance analysis, which is especially required in diagnostic applications.

## 8. Machine Learning for MIPs

The intervention of in silico molecular design with machine learning is accelerating the development of novel materials. Algorithms may be trained to generate thousands of hypothetical polymers based on the polymeric properties data and expertise from laboratory synthesis [153]. Furthermore, ML can enable prediction of characteristics of simulated polymers. Correspondingly, a recent study by Lowdon and coworkers tested ML algorithms for the assessment of binding affinities of MIPs to various molecular species [154]. Several algorithms were provided and applied to available experimental data to train the algorithms on the structures and binding affinities of various molecular species at varying concentrations. The optimum algorithm was identified based on the match of the predicted values and could be utilized for MIP affinity estimations.

As another example, AI models that can predict the folding and spatial structure of proteins [155,156] may be extended to analyze protein folding in monomer solutions. ML can innovatively assist the analysis of polymer variability based on the different conditions used. For example, if the algorithm is trained based on research that uses similar monomers but at different conditions of polymerization, ML can aid in reasoning the differences of the resulting polymers in terms of imprinting factors and binding capacities.

While the application of ML and AI for polymer science is currently only at the initial stages, their employment for MIPs may positively affect the dynamic development presently observable in predictive MIP design if the interdisciplinary involvement of material chemists and computer scientists is ensured.

## 9. Conclusions and Future Perspectives

The field of polymer chemistry is clearly evolving toward more sustainable synthesis practices. Computational design that minimizes the experiments needed for obtaining optimized structures and functions certainly assists these goals. As advanced software packages and computational resources are more readily available, accessible, and accurate, predictive modelling not only supports more targeted synthesis strategies but additionally limits the use of chemicals and minimizes environmental impact vs. conventional ‘trial-and-error’ approaches. While the combined application of QM, MM, and MD renders the simulation of complex macromolecular systems feasible, their full potential remains to be exploited in molecular imprinting technology. While rational MIP design is increasingly used, it is unquestionably not an all-encompassing solution. For some cases such as biological cell imprinting—or in general, complex biological species—it may remain impractical to use simulations. Alternatively, selected surface components of biological species that are being templated can be modelled along with their interactions using advanced computational capacities. Steps 4 to 6 of MIP optimization particularly demand more research efforts for simulating novel MIP constructs, material characteristics, and synthesis strategies including surface imprinting, solid-phase imprinting, etc. Computational models still yield idealized scenarios and predominantly homogenous polymers; yet, they may not be able to reveal the actual surface properties including morphology and/or pore size, which are obtained at the various experimental conditions. Nonetheless, the likewise continuous progress in modelling software packages and capabilities may allow an even more precise and rigorous simulation of macromolecular synthesis in the near future.

Using in silico techniques for experimental design and advanced synthesis strategies could lead to a more rational and traceable development of MIPs by devising standardized modelling protocols that are scalable for translation to an industrial level. In addition, novel polymerization strategies especially using, but not limited to, bio-based monomers are required to be integrated into virtual monomer libraries to screen against the wide range of target templates demanded by real-world application scenarios. Unlike the most commonly used acrylate-based monomers, alternative building blocks exhibit different characteristics and dynamics when dissolved, which requires innovative simulation strategies for optimizing the pre-polymerization steps. Since current modelling strategies have been demonstrated only for a limited set of monomers, solvents, and/or conditions, it will be necessary to test and validate advanced modelling protocols with alternative molecular building blocks. Likewise, the type of solvent models used during simulations—implicit, explicit, or hybrid—is indeed crucial for computationally studying the prevailing complex interactions. Consequently, simulations of the medium where MIPs are then deployed, and its effect on MIP performance, will be an interesting future area to explore. This may clearly be expanded towards cases including, but not limited to, contaminated water models or complex biological fluids.

MIPs remain among the most promising candidates for synthetic molecular recognition materials that may be experimentally and virtually tailored against a wide range of templates relevant for applications in environmental monitoring/sensing, food analysis, and clinical/medical diagnostics. However, with increasing dimensions of the template, the complexity in experimental and computational design likewise expands. Hence, for exceedingly ‘large systems’, currently a compromise between available computational resources and accuracy of predictive design has to be found and decided on for the most appropriate molecular modelling strategy in terms of obtained ‘resolution’, i.e., quantum, all-atom, coarse-grained, or mesoscale. The authors are convinced that only the smart interplay of different models will provide a sufficiently accurate mechanistic insight into the enabling of useful predictions that may in future be termed a ‘MIPs-by-design’ approach. The accelerating evolution of machine learning and artificial intelligence algorithms is clearly creating paradigm shifts in every science domain including material/polymer science; rational MIP design will certainly be among the beneficiaries of these developments, facilitating not only a significantly shortened ‘time-to-product’ but also supporting more sustainable synthesis strategies with minimal environmental impact.

## Data Availability

Data sharing not applicable.
